# LHON: Mitochondrial Mutations and More

**DOI:** 10.2174/138920211794520150

**Published:** 2011-03

**Authors:** E Kirches

**Affiliations:** Department of Neuropathology, Otto-von-Guericke University, Leipziger Str. 44, 39120 Magdeburg, Germany

**Keywords:** mtDNA, LHON, OXPHOS, cybrid, PTP, ROS, ATP.

## Abstract

Leber’s hereditary optic neuropathy (LHON) is a mitochondrial disorder leading to severe visual impairment or even blindness by death of retinal ganglion cells (RGCs). The primary cause of the disease is usually a mutation of the mitochondrial genome (mtDNA) causing a single amino acid exchange in one of the mtDNA-encoded subunits of NADH:ubiquinone oxidoreductase, the first complex of the electron transport chain. It was thus obvious to accuse neuronal energy depletion as the most probable mediator of neuronal death. The group of Valerio Carelli and other authors have nicely shown that energy depletion shapes the cell fate in a LHON cybrid cell model. However, the cybrids used were osteosarcoma cells, which do not fully model neuronal energy metabolism. Although complex I mutations may cause oxidative stress, a potential pathogenetic role of the latter was less taken into focus. The hypothesis of bioenergetic failure does not provide a simple explanation for the relatively late disease onset and for the incomplete penetrance, which differs remarkably between genders. It is assumed that other genetic and environmental factors are needed in addition to the ‘primary LHON mutations’ to elicit RGC death. Relevant nuclear modifier genes have not been identified so far. The review discusses the unresolved problems of a pathogenetic hypothesis based on ATP decline and/or ROS-induced apoptosis in RGCs.

## 1. INTRODUCTION

The first description of a patient suffering from Leber’s Hereditary Optic Neuropathy (LHON) dates back more than 150 years to a report of von Graefe [1], but the disease was first recognized as a distinctive clinical entity by the German ophthalmologist Theodor Leber in 1871 [2]. Due to a strong gender bias, favouring the outbreak of this inherited optic neuropathy in young men, it had been considered to be X-linked for a long time. Hundred years after Leber’s report, it was recognized that the mode of inheritance was maternal and thus compatible with mutations in the mitochondrial genome, the mtDNA [3]. In 1988, the first LHON-associated mtDNA mutation was discovered by Douglas C. Wallace and colleagues in the ND4 gene (NADH dehydrogenase 4), coding for a polypeptide of complex I of the electron transport chain (ETC) [4]. Since that time, five other complex I mutations have been confirmed to be clearly pathogenic in LHON (www.mitomap.org), a total of three mutations in polypeptides ND1 (G3460A) [5], ND4 (G11778A) [4] and ND6 (T14484C) [6] being responsible for 90-95% of all cases. A large number of other mtDNA mutations are suspected to cause LHON, to facilitate disease outbreak or modify the phenotype. However, it is a striking feature of the disease that all confirmed pathogenic mutations cause amino acid exchanges in complex I (NADH:ubiquinone oxidoreductase, E.C.1.6.5.3).

By a mechanism, which is yet not fully understood, these substitutions cause an acute oder sub-acute loss of retinal ganglion cells (RGCs) and their axons, comprising the optic nerve, leading to blindness or at least severe central vision loss. RGC loss occurs in about 50% of male and only 10-15% of female mutation carriers, but penetrance may vary even among pedigrees harbouring the same mutation. In contrast to several other mitochondrial disorders caused by defects of the bioenergetic machinery, LHON is often regarded as a non-syndromic disease, because RGC loss is the only clinically relevant phenotype in most patients. Only rarely other phenotypes have been ascribed to the same mutations besides optic atrophy, such as dystonia, multiple-sclerosis-like illness or cerebellar ataxia [7-12]. No other major neuronal losses have been detected in affected retinae, although e.g. the requirement of photoreceptor cells for ATP generation by oxidative phosphorylation (OXPHOS) is very high. The enigmatic selectivity of the phenotype is even more astonishing, if one keeps in mind that the frequent ND4 mutation often occurs in a homoplasmic state in blood and other tissues analyzed, comprising 100% of total mtDNA. Therefore, the amino acid substitutions seem to evoke a relatively mild biochemical defect in complex I, which leads to cell death only in RGCs. These cells exhibit a selective vulnerability for reasons, which are not fully understood. 

The disease can occur at any age, although most often young men in their second or third decade of life are affected. Usually both eyes are involved, but with a time delay of some monthes between them, which can be even longer than a decade in some cases [13]. Often the disease progresses rapidly leading to severe visual loss with only little probability of partial visual recovery [14]. A cecocentral scotoma develops with variable preservation of peripheral vision. Ophthalmologic findings are variable and major fundus changes may even be absent in the acute phase of the disease. Early ophthalmologic changes can include hyperhemic optic discs, disc pseudo-oedema and microangiopathy [15]. The disease finally leads to optic disc atrophy. 

Altough the primary causes of LHON have been identified in the last two decades to be amino acid substitutions in complex I polypeptides, this has not yet lead to a satisfying explanation of the pathophysiological mechanisms. The following two issues belong to the most disturbing enigmas of the pathomechanism:

- If bioenergetic failure should be the main consequence of the mutations, why are other OXPHOS-dependent retinal cells unaffected, such as photoreceptors ? Oxidative ATP generation relies on a functional ETC, which generates an electrochemical gradient across the inner mitochondrial membrane, and a functional ATP-synthetase, utilizing this gradient. Missense mutations (np8993) of the mtDNA-encoded ATP-synthetase subunit 6 (OXPHOS complex V) can cause a broader spectrum of retinal and brain dysfunctions and eventually cause lethal Leigh disease, characterized by brain necrosis (www.mitomap.org). These amino acid exchanges do not mainly induce RGC loss. What explains the selective vulnerability of RGCs towards the complex I mutations ?
- Why are male mutation carriers at a higher risk to develop LHON and which other factors trigger the disease ?

## 2. VARIABLE BIOCHEMICAL PHENOTYPE OF PRIMARY LHON MUTATIONS

Oxygraphic measurements of total oxygen consumption of whole cells, of mitochondrial respiration with complex I substrates, and assays of the NADH:ubiquinone oxidoreductase enzyme activity have been performed by many authors to characterize the biochemical phenotype of the three most common primary LHON mutations. Because they occurred in a homoplasmic state or at least accounted for a high percentage of total mtDNA in blood platelets and lymphocytes, these cells were often exploited as a starting material. Besides these ‘natural’ cell sources, Eppstein- Barr virus (EBV) transformed lymphoblasts were sometimes prefered to obtain larger amounts of starting material for biochemical analysis, or ‘transmitochondrial cybrids’ (see below) were constructed. 

Among the three common mutations, only G3460A (ND1) was invariably described to cause a considerable and significant decline of NADH:ubiquinone oxidoreductase activity [16-20]. In contrast to these results, no significant decrease of enzyme activity was observed in several studies analyzing the mutations in ND6 [21] or ND4 [16, 22], the latter accounting for most LHON cases. In other reports a minor decline of complex I activity was described in cells harbouring the ND4 mutation [20], or became only visible, if the statistical analysis was restricted to non-smokers [18]. This indicated that the influence of environmental factors may be large enough to mask the moderate effect of the mutation. Cell culture studies with cybrids revealed at least a decreased growth rate of mutated cells together with lower oxygen consumption [13]. 

A decline of mitochondrial respiration with complex I substrates (malate + pyruvat, malate + glutamate) was confirmed in assays of isolated mitochondria derived from EBV-transformed lymphoblasts and cybrids carrying any of the three common mutations [20]. These results, together with an aberrant behaviour of mutant mitochondria towards complex I inhibitors [19] clearly demonstrated a dysfunction of complex I in all cases. According to the heterogeneous enzymatic data and to the location of the amino acid substitutions in three different polypeptides, a precisely identical mechanism of the three common mutations was not likely. Nevertheless it was reasonable to assume a disturbed energy conserving function of complex I as the common result.

## 3. THE CYBRID MODEL

Since the discovery of the primary LHON mutations, the so called transmitochondrial cytoplasmic hybrid (‘cybrid’) model has been extensively used to characterize the biochemical phenotypes of mutations, independent of the nuclear background. To achieve this goal, Michael King and Guiseppe Attardi introduced a technique to deplete 143B.TK^**-**^ osteosarcoma cells of their mitochondrial DNA by sublethal concentrations of the DNA-intercalating dye ethidium bromide, and to repopulate these so called rho-0 cells with foreign mtDNA from enucleated ‘mtDNA donors’ [23]. For this purpose, the osteosarcoma cells had been exposed to ethidium bromide for a long time, until no residual mtDNA was detectable. Two viable rho-0 clones were obtained in this way, which were found to rely exclusively on glycolysis for ATP generation due to lacking OXPHOS. Moreover, the cells had developed pyrimidine auxotrophy due to the deficiency of the ETC-dependent dihydroorotate dehydrogenase [24]. Cell proliferation was strongly inhibited in the absence of uridine. After fusion of these cells with enucleated ‘cytoplasts’ of a cell line chosen to be the ‘mtDNA donor’, the generated cybrids were successfully repopulated with functional mitochondria and able to grow independent of uridine supplementation. The small percentage of successful viable fusions could thus be selected by a growth medium lacking uridine. If cytoplasts derived from LHON patients and controls are used as ‘mtDNA donors’ in this model, the biochemical alterations found in the resulting cybrids solely reflect the different mtDNA sequences. 

One of the original rho-0 clones obtained by King and Attardi (rho-0 206) has been distributed to several laboratories and many major LHON cybrid studies have thus been performed on the basis of a single 143B.TK^-^ rho-0 line [13, 22, 25-28]. These studies include importat work, which demonstrated a reduced rate of ATP synthesis and reduced ATP content in LHON cybrids under metabolic stress conditions (see below). It may be noteworthy that an additional risk can be seen in this restriction, besides the generally recognized problem of exploiting tumor cells as models for a defective neuronal energy metabolism. Techniques were developed to exploit other tumor cell lines for cybrid construction, such as teratoma or neuroblastoma lines, and to exploit blood platelets as ‘mtDNA donor cells’, which do not require enucleation. However, only a few LHON studies were performed with cybrids derived from the NTera-2 (NT2) teratoma line [29-31]. 

### 3.1. LHON Mutations Confer ATP Decline and Sensitize Cybrids to Apoptosis 

Due to the fact that RGC loss in LHON is painless and occurs without any signs of inflammation, an apoptotic cell death was suggested [32, 33]. Moreover, this mode of cell death was compatible with mitochondrial dysfunction. Danielson and colleagues reported enhanced apoptosis following FAS stimulation of cybrids bearing the ND1 or ND4 mutations, as compared to wild-type cybrids [25]. The mode of cell death was apoptotic by several criteria, including activation of caspase-3, increased DNA fragmentation (laddering) and enhanced annexin-V staining. Since the FAS-ligand is expressed in the retina, the authors suggested death receptor-mediated apoptosis to play a role *in vivo*. The authors proposed an enhanced opening propability of the mitochondrial permeability transition pore (PTP) due to complex I dysfunction as a potential mechanism for the enhanced sensitivity towards apoptotic stimulation, since ETC complex I had already been associated earlier with PTP function [34]. Although receptor-mediated apoptosis does not necessarily involve mitochondria, death receptor stimulation can be interconnected with these organelles *via *proteins of the Bcl2 (*B-cell lymphoma 2*) family. Fas-sensitivity may thus be enhanced in cells being sensitized to prolonged PTP opening. 

Shortly thereafter, Ghelli and colleagues demonstrated that cybrids with either one of the three most common primary LHON mutations undergo apoptotic cell death, as judged by chromatin staining and DNA laddering, when exposed to media, in which 20 mM glucose had been replaced by 5 mM galactose [26]. This effect was not observed within comparable time intervals in control cybrids. Galactose media were thought to facilitate the detection of OXPHOS deficiency, which may be masked by a rapidly running glycolytic pathway in tumor cells. Galactose, which is more slowly activated for glycolysis, was thought to render the cells more dependent on mitochondrial ATP generation, since only OXPHOS can satisfy the energy demand under conditions of a strongly restricted glycolytic activity. In OXPHOS-deficient cells ATP decline and eventually cell death should occur in galactose medium. The results of Ghelli and colleagues were thus interpreted in terms of enhanced apoptotic sensitivity of LHON cybrids due to bioenergetic failure, which cannot be observed in normal cell culture media due to compensation by glycolysis. These results are in accordance with apoptosis-induction in immortalized RGCs (cell line RGC-5) due to severe energy decline elicited by glucose deprivation [35]. 

Although galactose media were successfully used later in several important LHON studies, one critical remark should be considered. Galactose medium (5 mM) had been described in 1992 as a tool for a first screening of cells from patients suspected to suffer from mitochondrial disorders. Cells with several types of mutations affecting OXPHOS died in this medium, while cells with other types of OXPHOS-relevant mutations survived. Among the latter were LHON cells [36]. This suggests that the interpretation of all galactose effects in osteosarcoma cybrids, e.g. apoptosis induction, solely in terms of bioenergetic failure may be premature. 

**Fig. (1):**
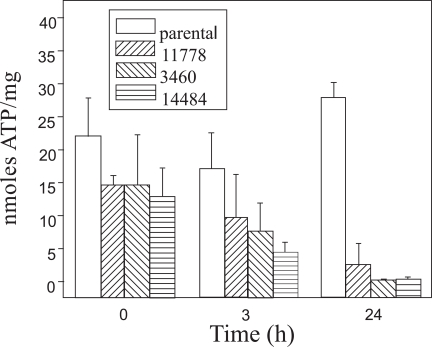
ATP content in 142B.TK^-^ cybrid cells, harbouring the three most common primary LHON mutations, and in the parental cell line were determined in triplicate after various incubation periods in galactose-medium. Modified according to [37].

Nevertheless, ATP decline (nmol ATP / mg cellular protein) occurs in 143B.TK^- ^LHON cybrids in DMEM with 5 mM galactose [27, 37]. This observation was made for cybrids containing any of the three mutations in polypeptides ND1, ND4 or ND6 (Fig. **1**), but did not occur in the parental cell line or control cybrids. The LHON cells rapidly died. In the study of Zanna and colleagues [27], the mode of cell death was analyzed in more detail. It was again demonstrated to be apoptotic, because cytochrome c, apoptosis inducing factor (AIF) and endonuclease G (endo-G) were released from the mitochondria into the cytosol. Because no caspase-activation was observed in this model, it was hypothesized that the concomitant dramatic decline in available ATP prevented the aggregation of a functional apoptosome, which requires ATP, while AIF and endo-G release are sufficient to execute apoptosis. 

Due to the incompletely understood role of galactose in generating metabolic stress, a direct proof was missing that the LHON mutations *per se* lead to an impaired ATP synthesis rate with complex I substrates. This was finally proven by Baracca and colleagues in 2005 [28], who detected the most severe impairment of the ATP synthesis rate, a 90% decrease, in cells bearing the G3460A mutation (ND1), which had been most consistently reported to mediate a severe decrease of NADH:ubiquinone oxidoreductase activity. 

### 3.2. Oxidative Stress, Calcium and Permeability Transition

Reactive oxygen species (ROS) are suspected to participate in the pathophysiology of several neurodegenerative diseases, such as Parkinsons Disease, Amyotrophic Lateral Sclerosis and Alzheimer’s Disease, and in the pathophysiology of mitochondrial disorders. Because ETC complex I is known to be a major ROS source, LHON mutations may increase ROS generation and thus enhance apoptotic susceptibility, eventually by favouring PTP opening. Calcium deregulation is another important factor, which is discussed in LHON. For example, dysfunctional mitochondria may be more prone to damage by increased cytoplasmic calcium concentrations. Calcium overload is well known to cause neuronal damage and plays a role for the opening probability of the PTP. 

**Fig. (2):**
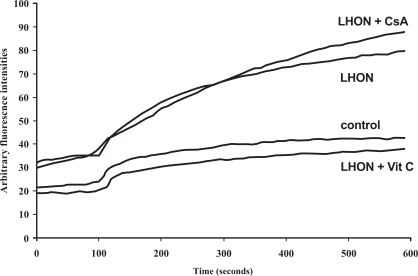
Representative graphs of DFF fluorescence visualizing intracellular ROS levels of NTera-2 (NT2) deruived LHON cybrids, carrying either the ND4 mutation at np11778 or normal mtDNA, over a period of ten minutes, using confocal real-timeimaging. After 100 seconds, H_2_O_2_ (100 µM) was added in order to saturate antioxidant defence mechanisms of the cybrids. It can be seen that Vit C (100 µM vitamin C) completely abolished the enhanced ROS levels of LHON cybrids. The PTP inhibitor CsA (cyclosporine A, 3 µM) was used in this set of experiments, since CsA had been found to partially inhibit calcium deregulation and cell death following stimulation of the LHON cybrids with an inhibitor of the smoth endoplasmic reticulum ATPase (thapsigargin). Since CsA did not influence DFF fluorescence, the measured ROS levels were not associated with permeability transition, which may occur after H_2_O_2_ addition. Modified according to [30].

Accordingly, there were good reasons to analyse the impact of externally applied oxidants and calcium on LHON cybrids. Already in 1997, Wong and Cortopassi described an enhanced sensitivity of 143B.TK^-^ LHON cybrids towards hydrogen peroxide, using a simple cell viability assay [38]. Interestingly, this sensitivity could be partially reversed by calcium withdrawal from the medium and by cyclosporine A (CsA), a known PTP inhibitor. These results pointed to a participation of oxidative stress and an altered PTP function in LHON. In a later study, the authors created NT2 teratoma cybrids, in order to generate a more realistic model, allowing neuronal differentiation by retinoic acid [29]. Only in the differentiated state, a significantly enhanced ROS production was observed in all LHON cells (mutations G3460A and G11778A) as compared to controls. This result may be interpreted in terms of elevated OXPHOS-dependency of the differentiated neurons in contrast to proliferating tumor cells, allowing to measure enhanced ROS-production by the mutated complex I. At least it demonstrated a role of the ‘neuronal environment’. Using confocal real-time imaging techniques, our collaborator F. Haroon and colleagues observed a slightly increased ROS level in the same LHON cybrids (G11778A) even without differentiation [30], which became much more pronounced following addition of an external oxidant (Fig. **2**). The cybrids had been kindly provided by G. Cortopassi and A. Wong (University of California, Davis, CA). 

In the same study, the authors investigated calcium deregulation, following a cytoplasmic calcium rise induced by acetylcholin and the inhibitor thapsigargin, which blocks calcium uptake into the endoplasmic reticulum. The cells were cultured in a temperature controlled chamber, which could be perfused with medium. Fluorescent real-time imaging could be performed with an inverted laser scanning microscope. The technique allowed to add or wash out acetylcholin and thapsigargin rapidly and to determine cytoplasmic calcium concentrations using cells loaded with a calcium-sensitive fluorescent dye (Fura PE3-AM). An incomplete reconstitution of normal cytosolic calcium levels, when the stimulated cells were washed with buffer, was observed selectively in LHON cybrids. This deregulation could largely be abolished by CsA, indicating a role of the mitochondrial PTP. The results suggested that mitochondria with mutated complex I have a lower ability to sequester calcium, because lower calcium loads are sufficient to elicit prolonged PTP opening (permeability transition). This could mean that normal cytoplasmic calcium concentrations may become toxic, if they occur in mutated neurons. 

**Fig. (3):**
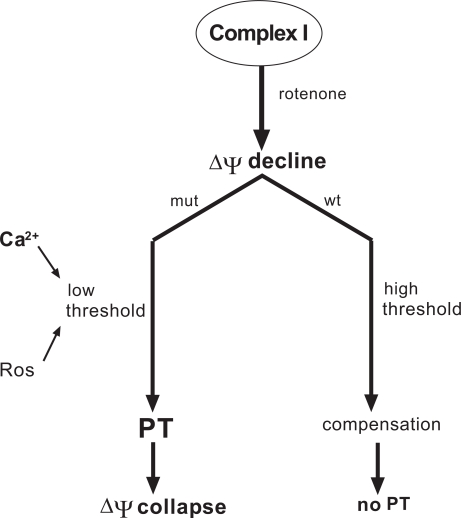
Schematic drawing outlining the hypothesis that LHON mutations in complex I (mut) may cause a shift in the potential, which is required for permeability transition (PT), towards the normal Δψ, creating an abnormally low voltage threshold for PT [39]. The shift is assumed to be mediated by Ca^2+^ deregulation and enhanced production of reactive oxygen species (ROS) in the mutant mitochondria, which both alter the permeability transition pore. As a consequence of this lowered threshold, a small rotenone-induced Δψ decline may immediately elicit PT leading to a complete and irreversible collapse of Δψ and oxidative phosphorylation. In cells with wild-type complex I (wt), the same rotenone-induced Δψ decline may not be sufficient for opening of the pore. Reconstitution of a normal Δψ may occur by a reversed action of the F_0_/F_1_-ATP-synthethase on the expense of glycolytic ATP (compensation).

The complex I inhibitor rotenone depolarizes the mitochondrial membrane potential in cybrids. Using cells harbouring the T14484C mutation (ND6) and a secondary ND6 mutation, it was recently shown that the divalent cation chelator BAPTA-TM, the vitamin E derivative Trolox and CsA were all able to abolish a rotenone-induced collapse of ∆ψ, which occurred in the mutant cells, but not in the parental wild-type cells after identical rotenone-treatment [39]. In addition, CsA was shown to partially abolish the depolarization caused by a replacement of the sugar glucose in the cell culture medium by galactose. The protective effects of CsA, Ca^2+^ withdrawal (chelators) and antioxidants in the mutant cells prompted the authors to propose a hypothesis, in which permeability transition (PT) causes the rotenone-induced collapse of ∆ψ in mutant cells, instead of being a result of it (for details see Fig. **3**). Porcelli and colleagues ascribed this central role to the PTP, since the pore is trigered by ∆ψ decline and the threshold voltage depends on Ca^2+^ concentration and ROS level. 

The authors hypothesized that the complex I mutations may shift the voltage threshold for PTP opening towards the resting level, due to Ca^2+^ deregulation and enhanced ROS generation. In healthy control cells, an initial rotenone-induced decrease of ∆ψ may be compensated on the expense of ATP, consumed by a reverse action of the F_0_/F_1_-ATP-synthetase. In cybrids with mutant complex I and lower voltage threshold for PT, the pore may open immediately after rotenone treatment, before the reversed ATP-synthetase has any chance to normalize ∆ψ. This compensatory process cannot occur anymore, once the inner mitochondrial membrane is permeabilized. Although there may remain doubts regarding this interpretation, the experiments again supported a role for oxidative stress, calcium and permeability transition in LHON. 

A potential role of oxidative stress was further underlined by some studies analyzing antioxidant defences. In the 143B.TK^-^ cybrid model, any of the three common mutations led to a fast increase in the oxidized form of glutathione (GSSG), if the cells were grown in galactose medium [40]. This was interpreted in terms of oxidative stress of mutant cells in a medium, in which they strongly rely on OXPHOS to satisfy their ATP demand. In the same cybrids cultured in normal DMEM, the oxidant *tert*-butyl hydroperoxide could induce apoptosis, but cells were protected by external administration of the antioxidant glutathione [41]. In our laboratory, NT2-derived LHON cybrids with the G11778A mutation, kindly provided by A. Wong and G. Cortopassi, had been found to contain decreased levels of total glutathione per mg cellular protein as compared to controls, if the cells had been treated shortly with the differentiating agent retinoic acid [31] in order to enhance their OXPHOS-dependency. 

Moreover, Qi and colleagues infected 143B.TK^-^ cybrids carrying the G11778A mutation with an adeno-associated viral vector (AAV) inducing mitochondrial overexpression of superoxide dismutase 2 (SOD2) in order to protect the LHON cells against enhanced superoxide production by the mutant complex I. The latter is known to deliver superoxide to the mitochondrial matrix compartment. Superoxide production decreased, as concluded from dihydroethidium fluorescence. The apoptotic index was reduced and short term cell survival enhanced [42]. The general ability of oxidative stress to induce apoptosis in RGCs has also been demonstrated in the RGC-5 cell line [43]. 

### 3.3. Limitations of Data Generated in Cybrid Models 

While an extremely high OXPHOS activity may be required especially in the unmyelinated axon sections of RGCs (see below), all cybrid models rely on the polyethylene glycol mediated fusion between cytoplasts or platelets (mtDNA donors) and rho-0 cells. The latter grow without OXPHOS and tumor cells in general use glycolysis besides OXPHOS even under aerobic conditions for their ATP supply. This means that artificial conditions, such as galactose medium, had to be found to simulate a high utilization of the mutant complex I. Cybrids prepared from NT2 teratoma lines were used as a model, which better reflects the neuronal phenotype, because NT2 cells can be differentiated by controlled cell density, medium conditions and retinoic acid. However, not all NT2-derived cybrids were responsive to the differentiating conditions. In responsive lines the achievable yield of terminally differentiated LHON-neurons was low [29], thus restricting the experiments to microscopic observations and other techniques not requiring larger amounts of cells. 

Moreover, tumor cell lines are often populations of genetically heterogeneous cells with non-identical sets of chromosomes and multiple chromosomal imbalances, visible on the molecular genetic level. Because the generation of rho-0 cells and cybrids represents a clonal selection from a tumor cell line, any clone will contain some nuclear genetic features, which were randomly picked up from the original cell population. In addition, genetic instability of the fusion products may lead to further diversification. The ideal model of identical nuclear background, allowing the isolation of mtDNA-mediated biochemical phenotypes, is not completely realized, as illustrated by occasional chromosomal losses and losses of heteroplasmy (LOH) in microsatellite markers [31]. Since a long term application of ethidium bromide was one of the most commonly used methods for mtDNA depletion, one may also ask, whether a large burden of chromosomal gene mutations is introduced into rho-0 cells. Besides differences on the DNA level, the transcriptome is strongly affected by the processes of mtDNA-depletion and cell fusion (cybridization), as clearly demonstrated by Danielson and colleagues, using a microarray approach [44]. As already suggested by other authors, the use of only a single cybrid clone with a particular mutation within a study bears risks. Clones derived from the same mtDNA donor sometimes vary remarkably in complex I activity. However, the strongest restriction of the existing literature data can be recognized in the use of a single rho-0 cell line for the central studies related to OXPHOS and apoptosis. 

## 4. MORE THAN BIOENERGETIC FAILURE?

As already stated above, a pathophysiologic hypothesis based solely on a decreased OXPHOS capacity of mutated RGCs is not completely satisfactory. 

Although there is as yet no precise hypothesis clearly addressing the issue of cell-type specificity, some interesting ideas have been formulated, which discuss potential contributions of the micromorphologic architecture of retinal nerve fiber layer (RNFL), optic nerve head and optic nerve. These hypotheses rely on the dramatic polarity of RGCs, with their cell bodies sending out axons of about 5 cm length to connect the neural retina with the brain [45]. This polarity includes an unequal distribution of mitochondria along the path of the axons and the fact that the axon sections within the RNFL are not wrapped into myelin sheets. 

The latter aspect was discussed by several authors as an explanation for the extremely high energy demands of this first axon section. The impossibility of saltatoric conduction requires a much higher ATP consumption by the Na^+^/K^+^-ATPase to maintain the excitability of the axolemma as compared to myelinated fibers [46]. An increased histochemical and immunohistochemical staining for the ETC enzyme cytochrom c oxidase (COX) had been observed within the optic nerve head as compared to the retrobulbar axon section within the optic nerve [47]. According to such staines, mitochondria (COX activity) seemed to be concentrated around the sites of major energy demand, as evidenced by simultaneous staining for voltage sensitive sodium channels [48, 49]. The unmyelinated state of the first axon section may thus contribute to an enhanced susceptibility of RGCs towards an OXPHOS decline. The important role of this first axon section for the process of neurodegeneration is nicely supported by the regional increase of RNFL thickness, observed early during desease development [50]. 

### 4.1. Possible Role of the Distribution of Mitochondria within the Axon

The interpretation of the gradient of mitochondrial content between the intra- and retrobulbar axon sections [47, 51-53] as a prerequisite of normal axon function has been the starting point for the hypothesis that any disturbance of this gradient may cause optic neuropathies [54]. 

When the axons leave the eye by a small opening in the sclera, they have to pass the lamina cribrosa, a perforated collagen plate. It had long been suggested that mechanical compression at this point restricts axoplasmic flow, leading to an accumulation of mitochondria in the prelaminar axon sections [33, 51, 52]. Small scleral openings had even been discussed as factors favouring optic neuropathies [55], although no change in axon diameter had been observed at the lamina cribrosa [52]. 

In the light of more recent histochemical and immunohistochemical findings, regarding the concentration of mitochondria at the sites of major OXPHOS demand, this hypothesis was replaced by its opposite: the physiological need for the observed mitochondrial density gradient [48, 53]. Histochemical stains confirmed the abrupt occurrence of myelin sheets in the postlaminar portion of the axons [48]. COX activity was not detactable in these sections, while it was high in the pre- and intralaminar regions. The results for COX activity were confirmed using antibodies directed against COX subnunits I and IV. This abrupt gradient in the distribution of COX subunits and enzyme activity was accompanied by an according gradient in the immunohstochemical staining for CNS-associated subtypes of voltage-gated sodium channels [48]. This coincidence indicates the physiological relevance of enhanced mitochondrial OXPHOS capacity near sites of the axolemma, where depolarization and subsequent ATP-consuming re-polarization are most required during nerve conduction. Precautions were taken to exclude the objection that the antibodies or secondary immuno-reagents may have a reduced access to axonal proteins in the myelin-covered areas. For this purpose, neurofilament immustaining was performed, which exhibited a strong signal along all portions of the axons under investigation. Such results can thus be interpreted in terms of a physiological need to trap mitochondria within those regions, which are unable of saltatoric conduction, instead of distributing mitochondria all along the axon with a homogeneous density. In rabbit eyes, where myelination starts at a more proximal site, enhanced mitochindrial activity is concomitantly shifted to this anatomical site. This view is further supported by the inverse relation between mitochondrial density and myelination along the optic nerve [47, 53]. 

Since axonal transport itself requires ATP, the maintainance of this gradient may be affected rather early by OXPHOS-relevant mutations. Inhibited axonal transport may thus contribute to a critical local energy deprivation at the sites, where most ATP is needed. Moreover, a reduced motility of mitochondria is expected to inhibit the dynamics of mitochondrial fission and fusion, which is necessary to exchange mitochondrial contents among these organelles and to avoid critical loss of components, including mtDNA. An exchange of components (mtDNA, RNA, soluble proteins, small molecules) between migrating mitochondria within the axon is thought to be necessary for the maintainance of a pool of functional mitochondria. This exchange may partially take place during short touches between the moving organelles, a mechanism designated ‘kiss and run’ [56]. 

### 4.2. Clues from OPA-1 Mutations

In some optic neuropathies with RGC loss, disturbed axonal flow and mitochondrial interaction have been discussed to participate in the pathophysiology. One of them, dominant optic atrophy (DOA) can be caused by mutations of the OPA-1 gene, coding for a dynamin-related GTPase, which is essential for structural features of the inner mitochondrial membrane (christae) and is required to maintain the ability of mitochondria to fuse with each other. While outer membrane fusion does not require OPA-1 in mammalian cells, a transient or complete mitochondrial fusion involving the inner membrane, and allowing intermitochondrial exchange of constituents, depends on OPA-1 [57]. Silencing of OPA-1 in HeLa cells resulted in a disorganized structure of the inner mitochondrial membrane, disturbed christae and fragmentation of the mitochondrial network [58-60], thus supporting the role of OPA-1 as a pro-fusion protein. The protein is further required for the interaction between mitochondria and cytoskeleton [61]. The view that a disturbed mitochondrial distribution along the axon, together with a disturbed intermitochondrial exchange may be an important cause of axonal degeneration, is further supported by studies of paraplegin-deficient mice [62]. Mutations in the gene (SPG7), encoding this protein, underlie a form of hereditary spastic paraplegia, which can include optic neuropathy. An impairment of axonal flow was demonstrated in these mice.

Also LHON mutations may favour a disturbed spatial distribution of mitochondria, which in turn may enhance bioenergetic decline at the sites of highest energy demand [63]. Some histopathologic features of LHON are compatible with disturbed axonal flow, such as clumps of mitochondria in the retrolaminar sections of remaining axons, accumulation of debris or microtubule depletion [63-65].

### 4.3. Glutamate Toxicity

One pathophysiologic hypothesis ascribes an important role in LHON to astroglial components of the neural retina. Having in mind the high mutational load observed in all investgated tissues, an indirect impact of a disturbed function of astroglial cells as metabolic supportes and protectors of RGCs appears to be possible. Complex I dysfunction in Müller glial cells was discussed to hamper the removal of glutamate from the neural retina, thus favouring glutamate excitotoxicity, which represents a major theme in neurodegenerative disorders. Müller glial cells are major glutamate sinks and their ability to pump glutamate largely, but not exclusively, relies on the activity of the excitatory amino acid transporter 1 (EAAT-1), designated glutamate aspartate transporter (GLAST) in rats and mice [66]. It has long been known that chronic low-dose glutamate administration into the vitreous is toxic to rat RGCs. Periodic glutamate injections into rat eyes, which increased the intravitreal glutamate concentration about 4-fold, were found to kill more than 40% of RGCs within three months [67]. EAAT-1 had been implicated in excitotoxicity within the retina [68]. Using the 143B.TK^-^ cybrid model, two cell clones for each of the mutations at np3460, np11778 or np14484 of the mtDNA were compared to controls with respect to expression and activity of EAAT-1. It had been previously established that this protein is the main glutamate transporter in osteosarcoma-derived cybrids. While expression was not affected by LHON mutations, transport activity was strongly decreased [69], a feature which was correlated with the level of ROS-production in these cybrids, but not with their ATP content. Redox-sensitive sulfhydryl residues are known to exist in the EAAT-1 transporter, which can form disulfide bonds, thereby down-regulating transpor activity [70]. This mechanism potentially links retinal excitotoxicity to oxidative stress induced by LHON mutations in Müller cells. The hypothesis was supported by the observation that the antioxidants Trolox and decylubiquinone could partially restore glutamate transport in LHON cybrids carrying the G3460A mutation [71]. The same report further supported an involvement of complex I deficiency in EAAT-1 dysfunction by demonstrating a reduced glutamate transport of control cybrids treated with the complex I inhibitor rotenone. In addition, partial complex I inhibition of primary rat retinal cultures by titration with rotenone, perturbed sodium-dependent glutamate transport [72], coincident with enhanced ROS production. However, the impact of EAAT-1/GLAST to mediate retinal glutamate toxicity remains questionable. Homozygeous GLAST knockout mice had normal retinae and other transporters were shown to participate in glutamate removal [73]. On the other hand, redox-sensitive thiol groups, regulating transport activity, were described in several glutamate transporters [70], which may theoretically be targets of oxidative stress. 

## 5. INSIGHTS INTO THE PATHOMECHANISM FROM NON-CYBRID STUDIES

### 5.1. Phosphorous MRS Reveals Altered Energy Metabolism in Non-Retinal Tissues

While cybrid studies of the energy metabolism were affected by the above mentioned constraints, a dysfunction of complex I at the level of enzyme activity had not been found consistently for all three common LHON mutations in patient tissues available for analysis. To assure a participation of energetic failure in the pathomechanism, the assessment of patient tissues with another, independent method was required to demonstrate a reduced phosporylation potential (PP) and a reduced energy reserve. ^31^P- phosphorous magnetic resonance spectroscopy (^31^P-MRS), a non-invasive spectroscopic technique, allows the relative quantification of the various phosphate-containing small molecules, which contribute to the PP, i.e. to the ability of cells to drive ATP-consuming biochemical reactions. Moreover, changes of the energy reserve, available to re-phosphorylate ADP, are reflected by peaks for creatinin phosphate (CrP). Several studies during the 1990s consistently observed a decline of these parameters in LHON patients or mutation carriers (G11778A) in brain [74, 75] and skeletal muscle [74-76]. These results correspond well with the observed decline of the energy-conserving functions of ND4-mutants in the cybrid model, despite the lack of major changes in complex I enzyme activity. 

A more recent study described a significant decrease of CrP and a corresponding increase of ADP for the G3460A mutation, but solely in occipital lobe tissue of the brain [77], not in skeletal muscle. Similar shifts of both parameters were not restricted to the index patient, but occurred also in two unaffected twin sisters The relative shifts of the ^31^P-MRS-peaks of the various phosphorous compounds *per se* can identify a disturbed energy metabolism. In addition, a quantitative estimation of PP according to the formula PP = [ATP] / ([ADP] x [Pi]) can be performed. For this purpose, an ATP-concentration around 3 mM was assumed and used to calibrate the peaks. The calculated PP was strongly reduced in the occipital lobes of the index patient and his unaffected sisters [77].

Taken together, these results strongly support an impaired energy conserving function of complex I in neural tissues of LHON patients and mutation carriers. The results support a role of decreased OXPHOS in the pathomechanism. Remarkably, these studies suggest that the basic biochemical defect occurs also in non-retinal neural tissues, which offers an explanation for the more complex LHON phenotypes, which are occasionally observed. 

### 5.2. Mitochondrial Dysfunction of RGCs Alone is Sufficient to Elicit LHON

Despite the more wide-spread tissue distribution of the biochemical defects, recent experiments with AAV-mediated *in vivo *gene transfer of the mutant human ND4-subunit into murine RGCs clearly demonstrated that the expression of the biochemical defect in RGCs is sufficient to elicit loss of these neurons and optic nerve degeneration [78]. 

Experiments of this type were thus not only a prerequisite to establish basic transfection techniques for an upcoming gene therapy approach (see paragraph 7). At the same time they provided the first direct *in vivo *evidence for the hypothesis that severe complex I dysfunction solely in RGCs is sufficient to elicit the disease, while concomitant expression of the defect in other neural or glial cell types of the CNS is not required. 

Since the retinal gene transfer experiments did not allow in-depth biochemical analysis, it may be argued that RGC death by overexpression of a mutant ND4-subunit may partially rely on unspecific toxicity due to a massive overload of cells with a foreign protein. However, Qi and colleagues [78] not only observed the expression of their transgenic ND4 polypeptide by Western blotting of retinal tissues, they also verified the import of the tagged polypeptide into RGC mitochondria by immunogold-labeling and demonstrated a distribution of the tagged mitochondria along the optic nerve. The authors also verified the incorporation of the human ND4-polypeptide into the mouse complex I by immunoprecipitation [78]. Finally, the objection of unspecific toxicity can largely be rejected, considering the successful abolishment of induced RGC loss by subsequent gene transfer of human wild-type ND4, as demonstrated by a recent study in rats [79]. 

## 6. ROLE OF MODIFIER GENES

**Fig. (4):**
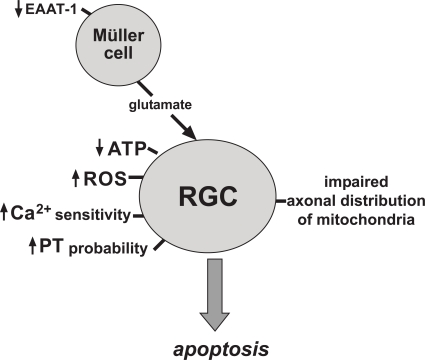
Schematic drawing showing the major pathophysiologic mechanisms discussed for primary LHON mutations to occur in retinal ganglion cells (RGC) or Müller glial cells. PT = permeability transition, EAAT-1 = excitatory amino acid transporter 1, ROS = reactive oxygen species.

Since all the above mentioned pathomechanisms (summarized in Fig. **4**) do neither explain incomplete penetrance, nor gender bias, additional genetic and environmental factors have been considered. Mitochondrial haplogroups have been discussed to play a role for the probability of disease outbreak in mutation carriers. A haplogroup represents a branch of the evolutionary tree of human mtDNA, which is characterized by a certain set of mtDNA polymorphisms. Haplogroup J was found to be over-represented in LHON pedigrees with the T14484C and G11778A mutations, suggesting a synergistic role of this haplogroup [80]. It may be hypothesized that a certain haplogroup more frequently carries additional polymorphisms in mitochondrial genes, which are associated with a lower OXPHOS capacity and favour LHON expression. For example, sub-haplogroups J2b and Jc1 were associated with higher penetrance of the G11778A and T14484C mutations, respectively. Both sub-haplogroups differ by one amino acid, encoded by the cytochrome b gene of the mtDNA (MTCYB), which may influence the function of ETC complex III. 

It is well known that the frequency of some haplogroups differs between ethnic groups. The importance of a given haplogroup-association may thus be restricted to certain geographic regions, as already stated by Hudson and colleagues [81]. 

Huge efforts have been undertaken in the last years to identify LHON susceptibility alleles in the nuclear genome. An X-chromsomal locus was in the focus due to the marked over-representation of males among the diseased carriers of primary mutations. An X-linked susceptibility locus had first been ascribed to the microsatellite DXS7 in Finnish pedigrees [82]. Several other X-linked loci were identified later, suggesting heterogeneity between ethnic groups [83-85], just as assumed for the role of mitochondrial haplogroups. In the year 2010, a genome-wide linkage analysis with 400 micro-satellite markers in LHON pedigrees from Thailand revealed a moderate association of LHON with markers not only on the X-chromosome, but also on chromosomes 1, 3, 12, 13 and 18 [86]. The region between 3q26.2 and 3q28 was most suggestive. Among other mitochondia-associated genes, it carries the gene OPA-1 and the gene *PARL* (*presenelin-associated rhomboid like*). The protease PARL is thought to cooperate with OPA-1 in influencing apoptosis. In the Thai pedigrees, a role of *PARL* as a susceptibility gene was further supported by the observation, that two intrageneous SNPs (single nucleotide polymorphisms) were LHON-associated. However, an independent study of Chinese LHON patients could not find any difference in the frequencies if these SNPs (alleles or genotypes) between LHON patients with a known primary mutation, suspected LHON patients without primary mutation and controls without optic neuropathy [87]. These conflicting results again support the notion that susceptibility loci determined in a geographically limited set of LHON pedigrees may bear the risk of specificity for a certain ethnic background. It may be considered that a set of susceptibility genes with heterogeneous allele distributions among ethnic groups may be involved in the disease. 

Many ‘environmental’ factors, such as smoking, alcohol abuse, the antibiotic ethambutol and environmental noxes had been discussed to play a role in LHON. Moreover, an impact of malnutrition and noxes was supported by the Cuban epidemic outbreak of a LHON-like optic neuropathy without any underlying mtDNA mutations [88]. However, only heavy smoking could be statistically verified to enhance the risk of mutation carriers to develop the disease [89]. 

## 7. FUTURE PERSPECTIVES OF LHON TREATMENT

Despite the incomplete knowledge of pathomechanisms, there is some hope for pharmacological and genetic intervention, which may slow down disease progression. 

Primary LHON mutations are single base substitutions, which require a high mutational load to cause the disease. Moreover, the degenerating retinal cell type (RGC) is exposed to the vitreous and thus easily accessible for plasmid or viral vectors injected into this compartment of the eye. These are prerequisites, which favoured the development of a local gene therapy approach, aimed to restore heteroplasmy by delivery of a corresponding wild-type subunit of complex I. On the other hand, several obstacles had to be overcome on this route. In living cells, mtDNA-encoded ND-subunits are transcribed and translated within the mitochondrial matrix and do not possess mitochondrial import sequences. Since efficient gene transfer into mitochondria is not possible *in vivo*, vectors had to be constructed for so called ‘allotopic expression’. The wtND-subunit of interest was cloned into vectors, which allowed regular cytoplasmic translation of the polypeptide and import into mitochondria by fusion to a suitable targeting sequence. To reach this goal, the cloned coding sequence of the ND-subunit had to be adapted to the ‘universal genetic code’, which slightly differs from the mitochondrial code. This was achieved by *in vitro* mutagenesis. Moreover, it was not clear in the beginning, if the allotopically expressed human subunit is correctly integrated into complex I during its assembly. 

A successful reversal of the ATP decline in G11778A cybrids was reported by allotopic wtND4 expression using an adeno-associated viral vector (AAV) [90]. Injection of the same vector, but carrying the mutant ND4 gene, into the vitreous of mouse eyes demonstrated for the first time that the route of gene transfer was applicable *in vivo*. Moreover, it suggested incorporation of the transgenic subunit into complex I, since optic neuropathy occurred [78]. An even more powerful proof-of-principle for the technique of gene delivery and for its therapeutic potential was recently provided by Ellouze and colleagues. The authors reported that RGC loss in rats cannot only be elicited by delivery of a mutant ND4 gene into rat eyes, but also be prevented by subsequent transfer of wtND4 two weeks later [79]. These developments created some hope regarding a possible gene therapy, which restores complex I function in patients with the most common ND4 defect [91]. 

Currently, pharmacological interventions with antioxidants represent a faster, and relatively safe route for developing LHON treatments, as evaluated in an ongoing double-blind, placebo-controlled clinical trial with the ubiquinone-derivative idebenone. However, these treatments are designed to attenuate a probable biochemical consequence of LHON mutations and not to cure the primary defect. The results of clinical trials will show, which impact idebenone may have for a better clinical outcome of patients. In all types of therapeutic interventions, an early start of treatment will be crucial, as will be the further improvement of methods, allowing early identification of mutation carriers at risk. Molecular genetic approaches will not be of major importance for the latter purpose, as long as no modifier alleles can be identified with an impact going beyond a limited set of regional pedigrees.
